# Astroglial calcium signaling displays short-term plasticity and adjusts synaptic efficacy

**DOI:** 10.3389/fncel.2015.00189

**Published:** 2015-05-27

**Authors:** Jérémie Sibille, Jonathan Zapata, Jérémie Teillon, Nathalie Rouach

**Affiliations:** ^1^Neuroglial Interactions in Cerebral Physiopathology, Center for Interdisciplinary Research in Biology, Collège de France, CNRS UMR 7241, INSERM U1050Paris, France; ^2^Université Paris Diderot, Sorbonne Paris CitéParis, France

**Keywords:** hippocampus, glia, neurons, neuroglial interactions, calcium signals, synapses, synaptic transmission, short-term plasticity

## Abstract

Astrocytes are dynamic signaling brain elements able to sense neuronal inputs and to respond by complex calcium signals, which are thought to represent their excitability. Such signaling has been proposed to modulate, or not, neuronal activities ranging from basal synaptic transmission to epileptiform discharges. However, whether calcium signaling in astrocytes exhibits activity-dependent changes and acutely modulates short-term synaptic plasticity is currently unclear. We here show, using dual recordings of astroglial calcium signals and synaptic transmission, that calcium signaling in astrocytes displays, concomitantly to excitatory synapses, short-term plasticity in response to prolonged repetitive and tetanic stimulations of Schaffer collaterals. We also found that acute inhibition of calcium signaling in astrocytes by intracellular calcium chelation rapidly potentiates excitatory synaptic transmission and short-term plasticity of Shaffer collateral CA1 synapses, i.e., paired-pulse facilitation and responses to tetanic and prolonged repetitive stimulation. These data reveal that calcium signaling of astrocytes is plastic and down-regulates basal transmission and short-term plasticity of hippocampal CA1 glutamatergic synapses.

## Introduction

Astrocytes are dynamic signaling elements able to sense, integrate and respond to synaptic activity. They are indeed equipped with a variety of channels, receptors and transporters allowing for detection of neuronal activity. They can also transmit information, in part by complex calcium signaling, to regulate in turn neurotransmission through multiple pathways (Perea et al., 2009; Pannasch et al., 2011; Rusakov et al., 2011; Dallérac et al., 2013; Bernardinelli et al., 2014).

Remarkably, astrocytes not only respond transiently to neuronal activity but can also display, like neurons, several forms of plasticity (Pirttimaki and Parri, 2013). These include a morphological plasticity of neuronal coverage during specific physiological conditions such as lactation or whisker stimulation (Iino et al., 2001; Genoud et al., 2006; Oliet and Bonfardin, 2010; Saab et al., 2012; Bernardinelli et al., 2014; Perez-Alvarez et al., 2014), as well as a functional plasticity of neuronal-induced currents or gliotransmitter release. For instance in the cerebellum, AMPAR-mediated calcium currents induced in Bergmann glia by stimulation of parallel fibers show specific activity-dependent short-term and long-term plasticity compared to adjacent Purkinje neurons (Bellamy and Ogden, 2005, 2006). Similarly in the hippocampus, astroglial potassium and glutamate uptake exhibit specific patterns of short-term plasticity (Manita et al., 2007; Sibille et al., 2014), as well as long term potentiation (Pita-Almenar et al., 2006; Ge and Duan, 2007; Zhang et al., 2009). Finally in the thalamus, astroglial glutamate release displays long term potentiation in response to cortical or lemniscal afferent stimulation (Pirttimaki et al., 2011). Such morphological and functional plasticity of astrocytes has been shown in various physiological contexts to regulate neuronal activity (Iino et al., 2001; Oliet et al., 2001; Pirttimaki et al., 2011; Saab et al., 2012; Sibille et al., 2014).

Among the responses evoked in astrocytes by neuronal activity, calcium signaling has been extensively studied and is thought to represent their excitability, since these cells are electrically non excitable. Astroglial calcium signaling can be mediated by multiple pathways, thought to generate signals with specific temporal and spatial patterns (Verkhratsky et al., 2012, 2014; Khakh and McCarthy, 2015). Numerous studies have shown that calcium release from endoplasmic reticulum internal stores via inositol triphosphate (IP3) receptors occurs after activation of plasmalemmal G-protein coupled receptors, such as metabotropic glutamate receptors (mGluRs; Verkhratsky et al., 2012). Such pathway is thought to underlie relatively slow calcium signaling in astrocytes. Additionally, calcium can also enter from the extracellular space via activation of membrane store operated channels, sodium-calcium exchanger or ionotropic receptors, such as NMDA, P2X or TRPA1 receptors in astrocytes from specific brain regions. These pathways may rather mediate fast and local signaling, possibly in astroglial perisynaptic processes (Verkhratsky et al., 2014; Khakh and McCarthy, 2015).

Nevertheless, how astrocytes encode calcium signaling and whether such signaling also displays activity-dependent changes remains elusive. Neuronal activity has been shown to modulate the frequency of hippocampal astroglial calcium transients mediated by mGluR activation (Pasti et al., 1995, 1997). In addition, astrocytes from the hippocampus or the barrel cortex can distinguish and preferentially respond to specific excitatory inputs in a nonlinear manner (Perea and Araque, 2005; Schipke et al., 2008). Yet, although astroglial calcium signals do correlate with the number of activated synapses, they were found not to be altered shortly after induction of hippocampal long-term synaptic plasticity, when detected with Fluo-5F, a calcium indicator with low calcium binding affinity (Honsek et al., 2012). Thus unlike other glial cells types such as NG2 cells or Schwann cells, where calcium signaling plasticity has been established (Ge et al., 2006; Bélair et al., 2010), whether astrocytes also show activity-dependent plasticity of calcium signals is still currently unclear.

In addition, activity-dependent calcium transients can induce various responses in astrocytes, including release of gliotransmitters, which can in turn modulate neuronal activity (Araque et al., 2014). Astroglial calcium signaling has been reported to modulate different regimes of neuronal activity, ranging from basal synaptic transmission to epileptiform events (Perea et al., 2009; Nedergaard and Verkhratsky, 2012; Dallérac et al., 2013; Araque et al., 2014). Contradictory results have nevertheless emerged about its role in synaptic transmission and plasticity (Fiacco et al., 2007; Petravicz et al., 2008; Agulhon et al., 2010), likely due to the distinct experimental manipulations used to increase calcium in astrocytes (Nedergaard and Verkhratsky, 2012). In fact whether these manipulations relate to physiological events is still an open question. Thus the role of endogenous astrocytic calcium signaling in physiological neuronal activity is currently matter of debate.

We here show that astroglial calcium signaling displays, simultaneously to excitatory synapses, short-term plasticity in response to prolonged repetitive and tetanic stimulations of Schaffer collaterals. We also demonstrate that acute and local inhibition of astrocytic calcium signaling increases glutamatergic synaptic transmission and short-term plasticity of Shaffer collateral CA1 synapses. These results reveal that astrocytic calcium signaling is plastic and can dampen transmission and short-term plasticity of hippocampal CA1 excitatory synapses.

## Material and Methods

### Animals

Experiments were performed on the hippocampus of wild type mice (C57BL6/J). Experiments were carried out according to the guidelines of European Community Council Directives of January 1st 2013 (2010/63/EU) and our local animal committee (Center for Interdisciplinary Research in Biology in College de France). All efforts were made to minimize the number of used animals and their suffering. Experiments were performed on the hippocampus of wild type mice. For all analyses, mice of both genders were used (PN15-PN22).

### Electrophysiology

Acute transverse hippocampal slices (400 μm) were prepared as previously described (Pannasch et al., 2012; Sibille et al., 2014) from 15 to 22 days-old mice. Slices were maintained in a storage chamber containing an artificial cerebrospinal fluid (ACSF) (containing in mM: 119 NaCl, 2.5 KCl, 2.5 CaCl_2_ 1.3 MgSO_4_, 1 NaH_2_PO_4_, 26.2 NaHCO_3_ and 11 glucose, saturated with 95% O_2_-5% CO_2_) for at least 1 h prior to recording. Slices were transferred to a submerged recording chamber mounted on an Olympus BX51WI microscope equipped for infra-red differential interference (IR-DIC) microscopy and were perfused with ACSF at a rate of 1.5 ml/min. Experiments were performed in the presence of picrotoxin (100 μM) and a cut was made between CA1 and CA3 to prevent the propagation of epileptiform activity. Extracellular field excitatory postsynaptic potentials (fEPSPs) and whole-cell patch-clamp recordings of astrocytes were performed in the CA1 *stratum radiatum* region of the hippocampus. The simultaneous recordings were carried out when intra-astroglial calcium chelation experiments (BAPTA, 10 mM) were made, and the field potential recording pipette, filled with ACSF, was placed in *stratum radiatum*, 20–50 μm away from the recorded astrocyte. Postsynaptic responses were evoked by stimulating Schaffer collaterals (0.1 Hz, stimulation intensity 10–20 μA) in CA1 *stratum radiatum* with ACSF filled glass pipettes. Paired-pulse facilitation was induced by delivery of two stimuli at an interval of 40 ms. Prolonged repetitive stimulation was performed at 10 Hz for 30 s, while post-tetanic potentiation was induced by stimulation at 100 Hz for 1 s in the presence of 10 μM of CPP ((Rs)-3-(2-Carboxypiperazin-4-yl-)propyl-1-phosphonic acid), to prevent induction of long-term potentiation. To investigate the effect of astroglial calcium chelation on evoked synaptic transmission or on short-term synaptic plasticity (PPF, PTP or responses to prolonged repetitive stimulation), fEPSPs were recorded in the same slice before and after calcium chelation in astrocytes. To do so and minimize mechanical movements in the slice, fEPSPs were first recorded while a BAPTA-containing patch pipette was sealed on an astrocyte (control condition), and the seal was then broken to chelate calcium only once fEPSPs were stable for at least 10 min. Experiments were independently performed (on different slices) for each experimental protocol (evoked synaptic transmission, PPF, PTP or prolonged repetitive stimulation). *Stratum radiatum* astrocytes were identified by their small somata, low input resistance and resting membrane potential, passive membrane properties (linear IV relationship), lack of action potential and extensive gap junctional coupling. Somatic whole-cell recordings were obtained from visually identified *stratum radiatum* astrocytes, using 5–10 MΩ glass pipettes filled with (in mM): 105 K-gluconate, 30 KCl, 10 HEPES, 10 phosphocreatine, 4 ATP-Mg, 0.3 GTP-Tris, 0.3 EGTA (pH 7.4, 280 mOsm), supplemented or not with BAPTA (10 mM). For intercellular dye coupling experiments, the internal solution contained sulforhodamine B (1 mg/ml), which diffused passively in astrocytes patched in current-clamp mode during 20 min. Recordings were acquired with Axopatch-1D or MultiClamp 700B amplifiers (Molecular Devices) digitized at 10 kHz, filtered at 2 kHz, stored and analyzed on computer using Pclamp10, Clampfit10 (Molecular Devices) and Matlab (MathWorks) softwares. Stimulus artifacts were blanked in sample traces. After acquisition data were binned at different frequencies according to the stimulation protocol used, and in order to clearly visualize the illustrated effects in each figure. Picrotoxin was obtained from Sigma, and all other chemicals were from Tocris.

### Calcium Imaging

All imaging experiments were performed simultaneously to electrophysiological field potential recordings in the same region of interest. Intracellular calcium measurements in astrocytes from hippocampal slices were made under single emission fluorescence microscopy using the fluorescent calcium indicator Fluo-4 AM (5 μM, Invitrogen), which has been demonstrated to load specifically astrocytes (Hirase et al., 2004). Loading was performed in ACSF with 0.02% Pluronic F-127 for 25 min in dark at room temperature. After recovery, slices were transferred to the recording chamber of an Olympus BX51WI microscope. Fluo-4 was excited at 488 nm through a light emitting diode (OptoLED, Cairn Research), controlled by the Axon Imaging Workbench software (Molecular Devices), triggering simultaneous acquisition of the electrophysiological recordings by Clampex10 software (Molecular Devices). Fluorescent light (>515 nm) emitted by labeled cells was detected with a long pass filter and an EM-CCD camera (Andor). Images were acquired at 4 Hz through a 20× water immersion objective (NA 0.95, Olympus) and stored on a PC. Images were processed and analyzed off-line with AIW imaging (Molecular Devices), Fiji (Image J) and Matlab (Mathworks) softwares. Background subtraction and bleaching correction were made in Fiji (Schindelin et al., 2012) and performed before fluorescence quantification. Background correction consisted in subtracting a filtered image that closely reflects the background to all of the time-serie images. This background image was obtained by applying a Gaussian blur filter with a 20-pixel radius to the first image of the serie. This filter also removed the acquisition noise on this first image, so that it doesn’t appear on the subtraction result images. The processed image serie was then corrected for photobleaching using the bleaching correction function in Fiji with the « Exponential fit » method. Data are then expressed as relative changes in fluorescence over baseline (ΔF/F0). After acquisition of images at 4 Hz, data were binned at different frequencies for clear visualization of the illustrated effects in figures. In average, 4–10 astrocytes were monitored per slice and several slices (*n* = 6) were monitored from at least 3 different animals.

### Statistics

All data are expressed as mean ± SEM and *n* represents the number of independent experiments. Statistical significance for within-group comparisons was determined by two-way repeated measures ANOVA, whereas two-tailed paired *t*-tests were used for between-group comparisons. Statistical analysis was performed in GraphPad Prism 6.

## Results

### Synaptically-Evoked Astroglial Calcium Signaling Displays Short-Term Plasticity

**Figure 1:**
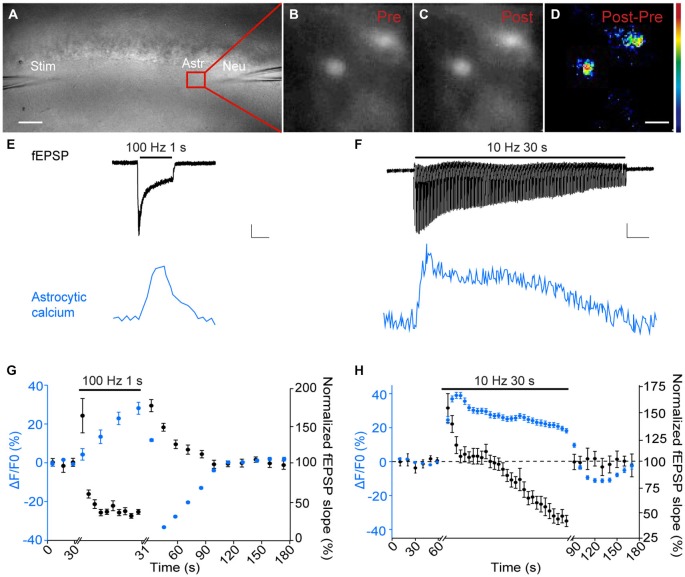
**Astroglial calcium signaling displays specific short-term plasticity patterns compared to synaptic responses. (A–D)** Sample pictures depicting in a hippocampal slice **(A)** dual recordings of fEPSPs (Neu), evoked by Schaffer collaterals stimulation (Stim) and astroglial calcium levels (Astr). Sample fluorescence images recorded in the red zone shown in **(A)** illustrating stratum radiatum astrocytes loaded with Fluo-4 AM before (Pre, **B**) and after (Post, **C**) tetanic stimulation of Schaffer collaterals (100 Hz, 1 s). The color-coded image illustrates the increase in astroglial calcium levels induced by tetanic stimulation (subtraction Post-Pre, **D**). Scale bars: 50 μm **(A)** and 10 μm **(B–D)**; color bar, 0–30% (ΔF/F0) **E,F**). Representative traces of simultaneously recorded fEPSPs (black) and averaged astrocytic calcium signals (blue) evoked by tetanic (100 Hz, 1 s, **E**) or repetitive (10 Hz, 30 s, **F**) stimulations. Scale bars: 0.2 mV, 5 ΔF/F0, 0.4 s **(E)** and 0.2 mV, 5 ΔF/F0, 2.8 s **(F)**. **(G,H)** Quantification of relative changes in astroglial calcium levels (blue) and fEPSP slope (black) induced by tetanic (*n* = 6 independent experiments with *n* = 35 astrocytes) **(F)** or repetitive (*n* = 6 independent experiments with *n* = 28 astrocytes) **(H)** stimulations, over baseline levels or responses measured before the onset of stimulations.

Glutamatergic synapses between Schaffer collaterals and CA1 pyramidal neurons in the hippocampus display several forms of short-term plasticity, such as post-tetanic potentiation (PTP) and facilitating and depressing responses to prolonged repetitive stimulation, which reflect alterations in presynaptic glutamate release. As calcium signaling in astrocytes is activity-dependent, we investigated whether it also exhibits some forms of short-term plasticity typical of excitatory synapses from CA1 pyramidal neurons. To do so, we recorded synchronously neuronal field excitatory postsynaptic potentials (fEPSPs) and astroglial calcium signaling in response to tetanic stimulation (100 Hz, 1 s) and prolonged repetitive stimulation (10 Hz, 30 s) of Schaffer collaterals (Figures 1A–D).

We found that astroglial calcium signaling displayed differential short-term plasticity patterns compared to adjacent pyramidal cells.

Tetanic stimulation of Schaffer collaterals (100 Hz, 1 s) results in PTP, a transient potentiation of excitatory transmission (Figures 1E,G). During the tetanus, astroglial calcium signaling continuously and gradually increased (peak increase (ΔF/F0): +28 ± 3%, *n* = 35 astrocytes from 6 slices), in contrast to neuronal responses, which only transiently potentiated (+61 ± 21%, *n* = 6) and then rapidly depressed (peak depression: −63.2 ± 8.2%, *n* = 6) (Figures 1E,G). In addition, after the tetanus astroglial calcium signaling rapidly exhibited a post-tetanic decrease (peak decrease (ΔF/F0): −32.5 ± 0.5% 20 s after the tetanus, *n* = 35 astrocytes from 6 slices) in contrast to neurons, which displayed a characteristic transient potentiation (fEPSP PTP: +74 ± 7.5% 10 s after the tetanus, *n* = 6) with a similar time course to that of the decrease of glial responses (Figures 1E,G). Remarkably, both neuronal and glial responses slowly came back to baseline levels ~1 min after the tetanus (Figure 1G).

Repetitive stimulation of Schaffer collaterals (10 Hz, 30 s) induces an initial facilitation of glutamatergic synaptic transmission, resulting from extensive glutamate release, followed by a depression, caused by depletion of glutamate vesicular pools (Figures 1F,H). As found for tetanic stimulation, astroglial calcium signaling exhibited a rapid and sustained increase during the whole repetitive stimulation (peak potentiation (ΔF/F0): +39.1 ± 1.8% after 3 s of repetitive stimulation, *n* = 28 astrocytes from 6 slices), in contrast to fEPSPs, which first transiently potentiated with stronger magnitude (peak potentiation: +52.8 ± 14.1% after 1 s of repetitive stimulation, *n* = 6) and then gradually depressed (peak depression: −57.8 ± 8% at the end of the repetitive stimulation, *n* = 6) (Figures 1F,H). Although both neuronal and glial responses reached their peak potentiation rapidly (1 s and 3 s, respectively) and then slowly decayed during the rest of the repetitive stimulation, the magnitude and time course of the responses differed, as astroglial calcium signal amplitudes reached a peak increase of ~ +40% within ~3–4 s of 10 Hz stimulation, and then slowly decayed to half peak responses after 30 s, while fEPSPs reached a peak potentiation of ~ +57% within ~1 s, fully decayed to baseline level after ~5–13 s and strongly depress (~ −35 to −60%) after ~20 s (Figure 1H). In addition, after the repetitive stimulation, the neuronal response returned immediately and steadily to baseline levels, while astroglial calcium signals exhibited a decrease in their amplitude below basal levels during ~1 min (reaching −11.1 ± 1.1% 60 s after the repetitive stimulation, and returning to basal levels 90 s after the stimulation, *n* = 28 astrocytes from 6 slices, Figure 1H).

Together, these data show that astroglial calcium signals display differential short-term plasticity patterns compared to neighboring excitatory synapses.

### Acute Calcium Chelation in Astrocytes Increases Evoked Excitatory Synaptic Transmission

**Figure 2:**
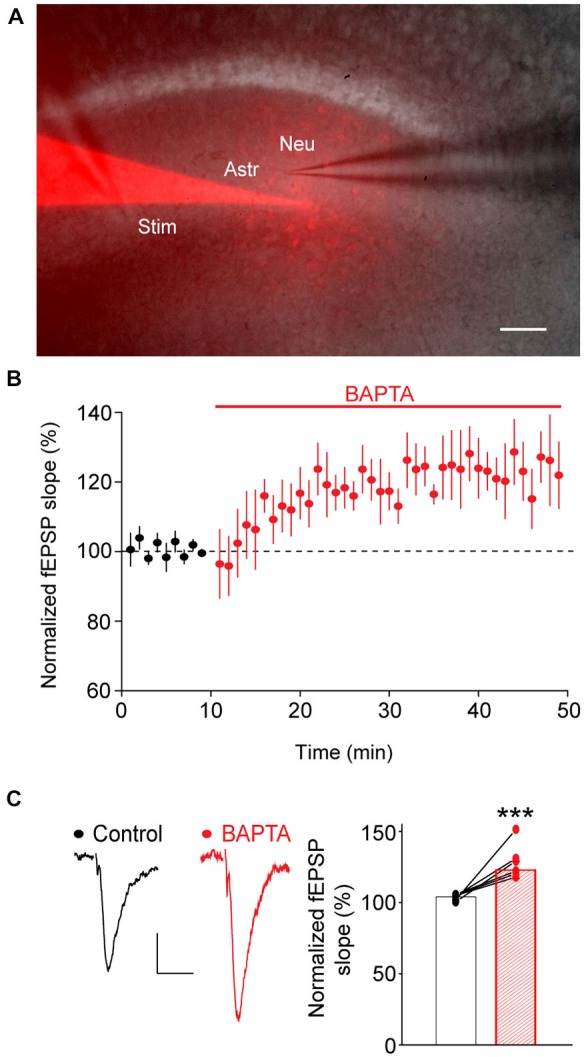
**Calcium chelation in astrocytes rapidly increases glutamatergic synaptic transmission. (A)** Sample picture of a hippocampal slice depicting dual recordings of fEPSPs (Neu), evoked by Schaffer collaterals stimulation (Stim), and a neighboring astrocyte (Astro) with a pipette containing BAPTA (10 mM) and sulforhodamine-B (red, 0.1%). Note the spread of sulforhodamine-B through gap-junction-mediated astroglial networks. Scale bar: 50 μm. **(B)** Quantification over time of fEPSPs evoked by a single stimulation of Schaffer collaterals in the same slices before (black) and after astroglial calcium chelation by intracellular BAPTA delivery (red, *n* = 6). **(C)** Sample traces and quantification of evoked fEPSPs recorded in the same slices before (black) and after 20–40 min of astroglial BAPTA infusion (red, *n* = 6). Asterisks indicate statistical significance (Student’s paired *t*-test, ***p < 0.001).

We then investigated whether in turn astroglial calcium signaling evoked by neuronal activity regulates locally the moment-to-moment glutamatergic synaptic transmission. To do so, we acutely and selectively inhibited calcium responses in local astrocytic networks, while simultaneously recording neighboring neuronal activity with fEPSP evoked by single stimulation of Schaffer collaterals. Calcium responses were specifically inhibited in astrocytes by intracellular dialysis of BAPTA (10 mM), a low molecular weight calcium chelator (astroglial calcium response peak amplitude: −70 ± 2%, *n* = 4 independent experiments on 26 astrocytes). BAPTA, together with sulforhodamine-B, a gap-junction permeable dye, were initially delivered to a single astrocyte via a patch pipette, and subsequently spread within populations of astrocytes through gap junction channels (Serrano et al., 2006; Rouach et al., 2008), as revealed by the extent of astrocyte dye coupling (Figure 2A). In all experiments, neuronal responses were recorded in close proximity (20–50 μm) to the astrocyte patched with the BAPTA-containing pipette. fEPSPs were initially recorded while the BAPTA-containing patch pipette was sealed on an astrocyte (control condition), and the seal was then broken to chelate calcium (BAPTA condition). fEPSPs were thus analyzed in the same slice before and after calcium chelation in astrocytes. In these conditions, we found that calcium chelation in astrocytes induced a rapid and sustained increase of ~20% in excitatory synaptic transmission (+22 ± 0.9% after 17 min of dialysis, *n* = 6, *p* < 0.001, Figures 2B,C). The increase occurred within ~10 min and lasted for at least 30 min. This effect was not due to leakage of BAPTA from the patch pipette in the extracellular space, because sealing an astrocyte with the BAPTA-containing patch pipette for 25 min had no effect on fEPSPs recorded prior to the astroglial seal (100.5 ± 1% of baseline response from 15 to 25 min after the seal, *n* = 5).

### Astroglial Calcium Chelation Potentiates Short-Term Plasticity Of Excitatory Synapses

To test whether astroglial calcium signaling also contributes to short-term synaptic plasticity, we performed the astroglial calcium chelation experiment while stimulating Schaffer collaterals with various patterns of activity. In all experiments, short-term synaptic plasticity was assessed in the same slice before and after astrocyte calcium chelation by recording activity-dependent changes in fEPSPs while the patch pipette was sealed on an astrocyte (control condition) and after 15 min of astrocyte dialysis with BAPTA (10 mM).

**Figure 3:**
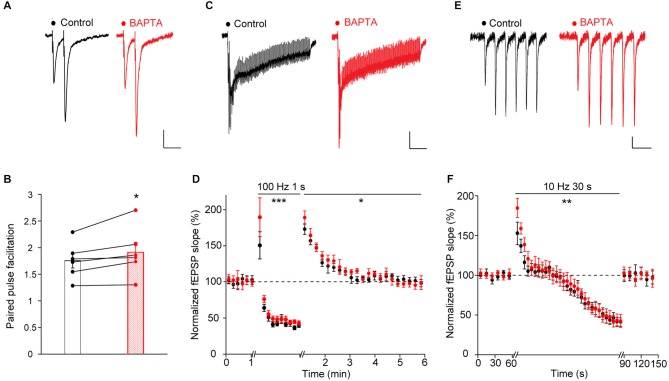
**Acute astroglial calcium chelation increases short-term synaptic plasticity. (A)** Sample traces and quantification of fEPSP paired-pulse facilitation (**A,B**, *n* = 6), post-tetanic potentiation (**C,D**, *n* = 10) and responses to repetitive stimulation (**E,F**, *n* = 6) recorded independently before (black) and after astroglial calcium chelation by BAPTA (10 mM, 20 min, red) in the same slices. Asterisks indicate statistical significance (**B**, Student’s paired *t*-test; **(D,F)**, two-way repeated measures ANOVA; *p < 0.05, **p < 0.01, ***p < 0.001).

Paired-pulse stimulation of Schaffer collaterals induced paired-pulse facilitation of excitatory synaptic transmission (PPF), a typical form of short-term plasticity in hippocampal CA1 pyramidal cells (Figures 3A,B). We found that calcium chelation in local astrocytic networks significantly increased PPF (PPF: before calcium chelation, 1.75 ± 0.13; 15 min after calcium chelation: 1.91 ± 0.18, *n* = 6, *p* < 0.05, Figures 3A,B).

Tetanic stimulation of Schaffer collaterals (100 Hz, 1 s) resulted in PTP, a transient potentiation of glutamatergic synaptic transmission (Figure 3D). Similarly to the effect observed on PPF, we found that the astroglial calcium chelation enhanced PTP (peak potentiation 10 s after the tetanus: before calcium chelation, +74 ± 7.5%; after calcium chelation: +90 ± 9.5%; normalized fEPSP 110 s after the tetanus: before calcium chelation, 105 ± 8%; after calcium chelation, 103 ± 4.4%; *n* = 10, *p* < 0.05, Figure 3D). Moreover, inhibition of calcium responses in astrocytes also potentiated the neuronal responses recorded during the tetanic stimulation (peak potentiation after 100 ms of stimulation: before calcium chelation, +50 ± 19%; after calcium chelation: +90 ± 27%; peak depression at the end of the tetanus: before calcium chelation, −65 ± 7.2%; after calcium chelation: −61 ± 6.8%; *n* = 10, *p* < 0.001), Figures 3C,D).

Prolonged repetitive stimulation of Schaffer collaterals (10 Hz, 30 s) induced a transient facilitation of excitatory synaptic transmission, followed by a depression (Figures 3E,F). As found for paired-pulse and tetanic stimulations, acute chelation of astroglial calcium increased the magnitude of the transient potentiation (peak potentiation after 1 s of repetitive stimulation: before calcium chelation, 52 ± 14.1%; after calcium chelation: 84 ± 11.8%, *n* = 6, *p* < 0.05, Figures 3E,F). However, amplitude of the maximal depression was unchanged (maximal depression at the end of the stimulation: before calcium chelation, −59 ± 6.5%; after calcium chelation, −57 ± 7.2%, *n* = 6, Figure 3F).

The effects of astroglial calcium chelation on the three forms of short-term synaptic plasticity described above were specific, as performing two consecutive times with a 15 min interval either paired-pulse, tetanic or prolonged repetitive stimulation of Schaffer collaterals did not alter by itself the induced short-term plasticities (PPF: 1.725 ± 0.09; 15 min after: 1.655 ± 0.006, *n* = 4, *p* > 0.05, Student’s paired *t*-test; PTP, peak potentiation 10 s after the tetanus: +75.50 ± 20.93; 15 min after: +72.29 ± 6.73, *n* = 5, *p* > 0.05, two-way repeated measures ANOVA; repetitive stimulation: peak potentiation after 1 s of repetitive stimulation: +59.1 ± 11; 15 min after: +60.4 ± 2.1; maximal depression at the end of the stimulation: −36.3 ± 4.4; 15 min after: −36.9 ± 5.7, *n* = 5, *p* > 0.05, two-way repeated measures ANOVA).

## Discussion

We here show that calcium signaling in hippocampal astrocytes displays several forms of activity-dependent short-term plasticity, with differential patterns compared to adjacent excitatory synapses. We also show that endogenous calcium signaling in astrocytes can down-regulate evoked glutamatergic synaptic transmission and short-term synaptic plasticity. These data provide novel insights into the dynamics of astroglial calcium signaling and its role in synaptic efficacy.

### Short-Term Plasticity of Astroglial Calcium Signaling

Activity-dependent plasticity refers to the strengthening or weakening of a response as a result of an increase or decrease in activity. Although this term is traditionally used for synapses, it is also currently used for glial cells, in particular for currents and calcium signaling from NG2 cells (Ge et al., 2006; Bélair et al., 2010), as well as for currents from astrocytes (Linden, 1997; Bellamy and Ogden, 2005, 2006; Pita-Almenar et al., 2006; Ge and Duan, 2007; Zhang et al., 2009; Sibille et al., 2014). Consistent with these findings, we here identified a novel type of astrocytic plasticity, i.e., an activity-dependent short-term plasticity of calcium signaling. We found two forms of calcium signaling short-term plasticity, which describe changes over time of astroglial calcium responses evoked by Schaffer collateral tetanic (100 Hz, 1 s) or prolonged repetitive stimulation (10 Hz, 30 s), which are stimulation patterns commonly used to induce specific forms of short-term synaptic plasticity (PTP or facilitating and depressing responses to prolonged repetitive stimulation, respectively).

Remarkably the two forms of plasticity were evoked by relatively low amplitude stimulation of Schaffer collaterals (10–20 μA), indicating that astrocytes display adaptive responses to moderate physiological synaptic activity. Interestingly, astrocytes were able to integrate relatively rapidly fast neuronal activity induced by either tetanic or repetitive stimulations, since astroglial calcium responses were evoked within hundred(s) of milliseconds. Moreover, both the short tetanic (1 s) and repetitive (30 s) stimulations evoked over time similar magnitude of intracellular calcium level increase in astrocytes, although the timing of the peak responses slightly differed (1 s vs. 3 s, respectively). This difference most likely reflects summation over time of evoked calcium elevations to reach peak amplitude. These data suggest that both types of stimulation may recruit similar intracellular pathway within slightly different timeframes.

In addition, both types of astrocytic plasticity displayed differential patterns compared to the short-term plasticity of synaptic responses. Although it is well admitted that astrocytes integrate neuronal activity through their calcium signaling, our data suggest that such signaling does not simply reflect neuronal responses, but exhibits specific behavior with proper dynamics (Volterra et al., 2014). Our results also suggest that during tetanic stimulation, astrocytes can integrate graded levels of presynaptic activity, through their calcium signaling, which likely does not saturate. In contrast, calcium signals may saturate in astrocytes after a few seconds of the 10 Hz stimulation, as they slowly decrease after 3 s.

The pathway underlying astroglial calcium signaling short-term plasticity was not identified in this study, but likely relies on activation of the canonical calcium release from internal stores induced by activation of glutamate metabotropic receptors. Indeed, both tetanic and prolonged repetitive stimulation of Schaffer collateral induce significant presynaptic release of glutamate, which spill over to likely activate the abundantly expressed mGluR5 in neighboring astrocytes from juvenile mice (Sun et al., 2013; Verkhratsky et al., 2012). This pathway has already been shown in astrocytes to be recruited in a graded manner during Schaffer collateral stimulation, correlating with the number of activated synapses (Honsek et al., 2012). In addition, the time needed to reach maximum amplitude of mGluR-mediated calcium responses in astrocytes is relatively slow, i.e., in the range of seconds (1–3 s) (Honsek et al., 2012), as reported in our study. Finally, the slow decrease in calcium signaling that occurs after a few seconds of the 10 Hz stimulation may reflect desensitization of mGluRs as well as gradual depletion of internal calcium stores during the course of the stimulation. We cannot however exclude the involvement of other molecular pathways mediating extracellular calcium entry in astrocytes through store operated channels, sodium-calcium exchanger or ionotropic receptors, such as P2X or TRPA1 receptors, which may underlie fast signaling initiated in astroglial perisynaptic processes that slowly propagate to cell soma (Verkhratsky et al., 2012, 2014; Khakh and McCarthy, 2015).

Remarkably, astrocytes did not only show a differential pattern compared to neurons during the tetanic and repetitive stimulations, but also after. Indeed, while postsynaptic neuronal responses displayed a typical post-tetanic potentiation, astroglial calcium signals showed a mirror response with a post-tetanic decrease, slowly coming back to baseline levels within a minute, akin to neuronal responses. Similarly astroglial calcium signals showed a decrease after the repetitive stimulation, in contrast to neuronal responses which returned immediately to baseline levels. Such post-tetanic and post-repetitive decreases in astroglial calcium signaling resemble the depression of the synaptically-evoked potassium and residual currents (I_K_ and I_res_) that we recently identified in hippocampal astrocytes after tetanic and repetitive stimulations (Sibille et al., 2014). They are also reminiscent of typical undershoot responses (such as extracellular potassium levels and space volume) occurring after similar high frequency stimulations (D’Ambrosio et al., 2002; Chever et al., 2010; Haj-Yasein et al., 2011; Pannasch et al., 2011; Bay and Butt, 2012). These short-term plasticities may represent an adaptive response to prevent prolonged activation of astroglial calcium signaling, thus enabling local and transient increase in astroglial signaling during the stimulations. They may also serve to increase the signal-to-noise ratio by contrasting the astroglial calcium responses. In addition, the short-term decreases in astroglial calcium signaling might also code for downstream intracellular pathways, which would regulate in turn neuronal activity. The mechanism underlying the transient depression of astroglial calcium signaling after tetanic and repetitive stimulations still needs to be investigated. It could result from the combination of mGluRs desentization, intracellular calcium store depletion and astroglial calcium release through calcium ATPases, sodium-calcium exchangers or connexin and pannexin hemichannels, which can be activated by increase in cytosolic calcium (Verkhratsky et al., 2012; Cheung et al., 2014).

### Regulation of Neurotransmission by Endogenous Calcium Signaling

Astroglial calcium signaling is generally thought to modulate neuronal activity through multiple and complex pathways (Volterra et al., 2014). Although many studies have shown that calcium signaling in astrocytes regulates various regimes of neuronal activity (Perea et al., 2009; Nedergaard and Verkhratsky, 2012; Dallérac et al., 2013; Araque et al., 2014; Volterra et al., 2014), some failed to detect any effect on synaptic transmission or plasticity (Fiacco et al., 2007; Petravicz et al., 2008; Agulhon et al., 2010). These discrepancies led to the view that calcium signaling in astrocytes is more complex than initially thought, and that different effects may occur according to the manipulations used to alter astroglial calcium. Indeed to interfere with astroglial calcium signaling, several approaches have been used, including acute downregulation using intracellular calcium chelation with BAPTA or calcium-clamp, chronic and targeted inhibition using transgenic mice with impaired astroglial store-mediated calcium signaling (IP3R2^−/−^ mice), as well as acute activation using pharmacogenetic approach to activate specifically calcium signaling in astrocytes in a transgenic mice expressing in astrocytes in an inducible manner a Gq-G protein coupled receptor (MrgA1) not expressed in the brain and activated by a brain exogenous ligand (Fiacco et al., 2007; Petravicz et al., 2008; Agulhon et al., 2010; Henneberger et al., 2010; Nedergaard and Verkhratsky, 2012; Volterra et al., 2014). Identifying the manipulations that relate to physiological events or interfere with endogenous signaling is a current effort to unravel the actual role of astroglial calcium in physiological processes.

We here aimed at clarifying such role on hippocampal physiological synaptic transmission and short-term plasticity. To do so, we used calcium chelator infusion into populations of astrocytes through patch pipettes to downregulate acutely calcium signals in astrocytes. Remarkably, we found that such manipulation relatively rapidly unleashed evoked excitatory synaptic transmission and several forms of its short-term plasticity. Using similar approaches, several studies have shown *ex vivo* or *in vivo* during alternative paradigms that acute downregulation of endogenous calcium signaling, via calcium chelation with BAPTA or calcium-clamp (Henneberger et al., 2010), regulates physiological synaptic activity such as basal synaptic transmission (Panatier et al., 2011), spontaneous postsynaptic currents and evoked bursts (Benedetti et al., 2011), LTP (Henneberger et al., 2010), heterosynaptic depression (Serrano et al., 2006), short-term synaptic depression (Andersson and Hanse, 2010), as well as cholinergic-induced LTP (Navarrete et al., 2012). Noteworthy in most cases, astroglial calcium signaling was found to increase neuronal activity through gliotransmitter release (Serrano et al., 2006; Henneberger et al., 2010; Panatier et al., 2011; Navarrete et al., 2012). However, each regulation was reported to be mediated by a different gliotransmitter or targeted receptor, such as D-serine (Henneberger et al., 2010), glutamate (Navarrete et al., 2012) or ATP, subsequently metabolized into adenosine and activating either presynaptic A1 (Serrano et al., 2006) or A2 receptors (Panatier et al., 2011). We here found that calcium chelation in astrocytes increased evoked excitatory synaptic transmission, paired pulse facilitation, post-tetanic potentiation and responses to repetitive stimulation in the hippocampus, implying that in these cases astroglial calcium signals downregulate synaptic transmission and short-term plasticity.

However, the underlying mechanism still remains to be determined. These astroglial regulations of synaptic efficacy may be directly mediated by calcium-dependent release of inhibitory gliotransmitters, such as ATP-derived adenosine activating A1 receptors, cannabinoids activating CB1 receptors (Navarrete and Araque, 2008), or GABA (Angulo et al., 2008) acting on GABA_A_ or GABA_B_ receptors, pathways all well known to decrease presynaptic glutamate release. However other mechanisms may be at play, such as calcium-dependent potassium uptake through astroglial Na/K ATPase, leading to decreased extracellular potassium levels and thereby reduced excitatory synaptic transmission, as recently reported in the hippocampus (Wang et al., 2012). Alternatively calcium-dependent astroglial synapse coverage, promoting at the synapse tight morphological interactions (Iino et al., 2001; Saab et al., 2012; Tanaka et al., 2013; Bernardinelli et al., 2014) and efficient astroglial clearance of glutamate (Pannasch et al., 2014) or likely potassium, as well as availability of astrocyte-derived neuroactive factors, might also dampen glutamatergic transmission. Interestingly our results are comparable to the ones obtained in two recent studies, showing that calcium chelation in the barrel cortex increased the frequency of spontaneous excitatory postsynaptic currents, as well as the amplitude and duration of evoked bursts (Benedetti et al., 2011), while in the hippocampus it inhibited a post-burst short-term depression of glutamatergic transmission (Andersson and Hanse, 2010). In both studies the underlying mechanism was not identified, although D-serine, adenosine or glutamatergic transmission were not found to be involved in regulation of neuronal activity from the barrel cortex (Benedetti et al., 2011). It is thus tempting to speculate that the differential effects of astroglial calcium chelation on neuronal activity may rely on the type of neuronal activity, the brain area and the developmental stage studied, which are likely associated with different forms of astroglial calcium signals and downstream intracellular pathways. Such multiple effects illustrate the complexity of the astroglial calcium signaling code, which remains to be further unraveled.

## Conflict of Interest Statement

The authors declare that the research was conducted in the absence of any commercial or financial relationships that could be construed as a potential conflict of interest.
